# Potential for Use of Recycled Cathode Ray Tube Glass in Making Concrete Blocks and Paving Flags

**DOI:** 10.3390/ma15041499

**Published:** 2022-02-17

**Authors:** Dušan Grdić, Iva Despotović, Nenad Ristić, Zoran Grdić, Gordana Topličić Ćurčić

**Affiliations:** 1 Faculty of Civil Engineering and Architecture, University of Niš, Aleksandra Medvedeva 14, 18000 Niš, Serbia; nenad.ristic@gaf.ni.ac.rs (N.R.); zoran.grdic@gaf.ni.ac.rs (Z.G.); gordana.toplicic.curcic@gaf.ni.ac.rs (G.T.Ć.); 2 Faculty of Mechanical and Civil Engineering in Kraljevo, University of Kragujevac, Dositejeva 19, 36000 Kraljevo, Serbia; ivickad@gmail.com

**Keywords:** concrete blocks, concrete flags, recycled cathode ray tube glass, mechanical characteristics, radioactivity, leaching test

## Abstract

The potential to use waste glass, including cathode ray tube (CRT) glass, for making new products or as an admixture to existing ones is being intensively investigated. This kind of research intensified particularly in the period after CRT TV sets and computer monitors were replaced in the market by the advanced technology of thin film transistor (TFT) and liquid crystal display (LCD) screens. Cathode ray tube glass represents a considerable part of electronic waste (e-waste). E-waste globally increases at a far higher rate than other solid waste materials. There is a possibility to recycle cathode ray tube glass and use it in the construction industry. This paper shows the test results of physical and mechanical properties of blocks and paving flags. The reference specimen was made with quartz sand, while the other product employed a combination of quartz sand and ground panel cathode ray tube glass. The glass was ground to the fraction 0.25/1.00 mm, which corresponds to quartz sand fineness. The following tests were performed: shape and dimensions, resistance to freeze/thaw and de-icing salts, water absorption, splitting tensile strength and tensile strength by bending. Special attention was paid to the tests of Böhme wear resistance, slip resistance of the top surface of CRT products using a pendulum, radioactivity and leaching. The texture of the experimental concrete products was observed by SEM (scanning electron microscopy) and analyzed. The results obtained by experimental testing unequivocally show that CRT glass can successfully be used for making concrete blocks and paving flags.

## 1. Introduction

**Figure 1:**
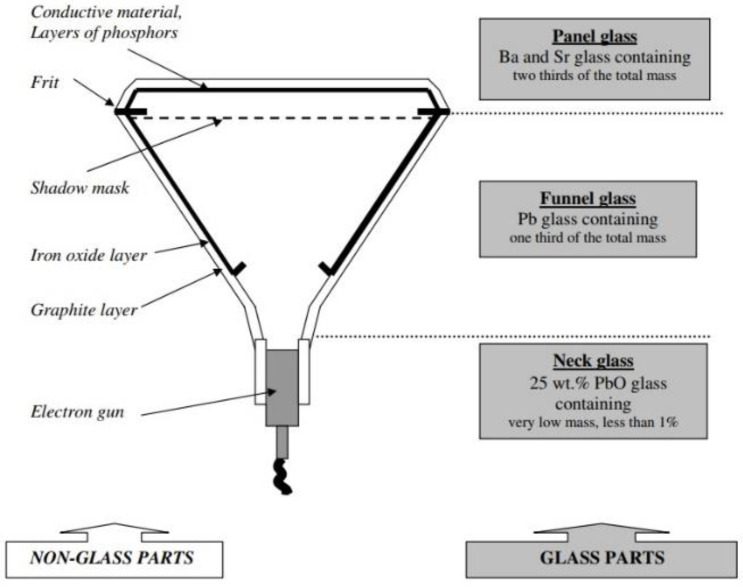
Appearance of cathode ray tube and its constituent parts [6].

Computer and TV screens with cathode ray tubes (CRTs) have not been sold in Europe since 2011, when, through technological advancements, they were replaced by new TFT (*thin film transistor*) and LCD (*liquid crystal displays*) screens. Considering the “service life”, i.e., the duration of the cathode ray tube screens, and the fact that they can still be found in households, it is clear that a considerable quantity of these devices is stored in landfills. Precise data on the amounts of generated waste CRT glass are not readily available. According to the data shared by the WEEE Forum, the amount of CRT displays generated in the Europe (30 countries) has been declining since 2009, and it is estimated that in 2020, this amount was around 250 kt [1]. In the USA, there are around 232 million CRT devices which are still being used, so considerable amounts of those devices end up in landfills each year—it is believed that around 85% of these devices will be collected in the next ten years. According to the data in Ref. [2], in the USA in 2018 alone, 500 kt of CRT waste was generated. In addition, Shaw Environmental Inc. (Hamilton Township, NJ, USA) estimated that around 3.2 million units of CRT technology would require proper waste management every year as it will reach its end of life in 2022 [2]. TV screens constitute the largest part of electronic waste in China, with around a 50% share. According to the research of Quinghua University, the total amount of collected waste CRT glass exceeds 5.2 million tons, with 3.5 million tons of tinted panel glass, 1.7 million tons of funnel glass and 0.7 million tons of black-and-white glass. However, due to the high cost of CRT glass recycling and low value of the obtained raw material, only a fraction of waste glass is recycled in China in a formal way, observing all the regulations protecting human health and the living environment [3]. According to Singh et al. [4], the estimated waste generation of CRT TVs in China in the period 2010–2020 is around 3240 kt. In Serbia, in 2016, around 3080 tons of CRT glass were collected. The current practice in Serbia is that the collected CRT glass is exported to other countries and the countries taking in the recycled CRT glass are paid for that [5]. The three basic glass elements of a CRT screen are neck glass, funnel glass and panel glass (Figure 1). Neck glass contains around 25% lead, which is far more than other parts. Funnel glass is the largest part of the cathode ray tube, and it contains around 20% lead. The panel is the visible front part of the cathode ray tube which contains almost no lead (0–3%). It is coated with barium and strontium layers whose role is to protect viewers from the adverse effects of UV and X-ray radiation. The funnel and panel are mutually joined by the frit.

At the start of the CRT glass recycling process, it is necessary to separate glass by types, because the different chemical composition of each type of glass in one cathode ray tube demands a different recycling method. It is standard to first breach the neck of the cathode ray tube to decompress the inside of the tube. Glass parts are separated in several ways: using hot band, diamond saw, jet or laser cutting [7]. Considering the problematic chemical composition of cathode ray tube glass (presence of lead), which constitutes the largest part of every screen, the transport and storage of such waste must meet special conditions. Therefore, from the environmental aspect, the recycling process of CRT glass is very important, but also complex—since there is no production of new CRT devices, there is no production process to which the old devices can be returned. The only option is to use the waste glass in new products. Over the past decade, extensive research was conducted on the possibilities of using waste glass in the production of crystal and radioactive glass, ceramic flags, artificial marble, glass jewelry, transparent lead coatings, sand–cement bricks, cement mortar and, to a smaller extent, concrete and concrete prefabricates.

The idea to use glass to make concrete is not new [8,9]. When making cement mortar, waste glass can be used as a filler, i.e., as a substitution for a certain amount of natural aggregate [10,11], most often as a replacement of a part of cement [12,13,14,15], or in road bases and sub-bases [16]. Researchers have investigated various types of waste glass: panel and funnel glass of CRT screens, decorative crystal glass, fluorescent lamp glass, glass food containers, facade glass, glass cullet, glass sludge (a side product of polishing and processing glass) and, in recent times, the glass from TFT–LCD screens. Hui Zhao et al. [17] tested the properties of mortar where a part of natural river aggregate was replaced by CRT funnel glass. The percentages of glass used to replace the fine river aggregate were 0%, 25%, 50% and 75% by mass. Mortars with added glass had a higher strength than the reference mortar at all ages. One research group explained this by the enhanced packing of the aggregate grains and with the assumption that the presence of CRT aggregate in mortar accelerates the cement hydration. Tensile bending strength was tested at the same mortar ages as the compressive strength. With the increase in the share of recycled glass in mortar, there was an increase in the strength of the mortar. The same authors proved that the inclusion of CRT glass sand in the concrete could also reduce the drying shrinkage of concrete and increase the alkali–silica reaction (ASR) expansion of the mortar. Gerry Lee et al. [18] determined that with the increase in the percentage of recycled glass replacing natural aggregate, irrespective of the fineness, there is a reduction in the density of concrete, which is explained by the lower specific gravity of glass in comparison to the fine natural aggregate. The research of Eshmaiel Ganjian et al. [19] included different recycled materials and the verification of their potential to be used in the production of concrete blocks. Materials used were granulated slag, dust from the cement production process, recycled gypsum panels, fly ash from chimneys and thermal power plants, metal dust from the iron alloying process and waste glass. Tensile splitting tests at the ages of 14 and 28 days determined that only the series with granulated slag and dust from the cement production process met the standard EN 1338 and durability requirements. Dondi et al. [20] tested the potential of the use of CRT glass for the production of bricks and roof flags. The experimental results indicate that both types of CRT glass have an impact on the reduction in the plasticity of unfired products and on the increase in shrinkage during drying. Andreola et al. [21] researched the potential of using CRT glass in the production of ceramic glazing. Glazing with CRT glass reduces the potential environmental hazard by 36% compared to ordinary glazing. Ceramic foam is most often used for thermal insulation and soundproofing as well as for absorption of harmful particles in the air. Ref. [22] indicated that one can successfully produce this foam with the addition of CRT glass. The use of conical glass for the production of crystals would reduce the addition of lead–oxide by 0.2 tons. Yu et al. [23] used cleaned funnel CRT glass for the production of fluorescent lamps with low lead content. Thung-Chai Ling et al. [24] researched the potential of using mortar with the addition of glass in radiology as an absorber of harmful X-rays. The best absorption of X-rays was observed in the mortar series in which the total amount of aggregate was replaced by CRT glass with a high content of lead. In the case of the sample 5 mm thick, 56.2% lower radiation was measured compared to a reference specimen of the same thickness. Such a result can be explained by the fact that CRT glass with lead has a higher density than natural aggregate and that its atomic structure acts on X-rays by reducing their energy and penetration depth.

Based on the review of a number of papers, it can be concluded that when CRT recycled glass is used to replace part of the natural aggregate in concrete, strength and tensile strength by bending are better, drying shrinkage can be reduced or increased (the results differ) and density is decreased. It is also concluded that few studies are based on concrete precast elements made with CRT glass. The subject of this paper is the production of concrete paving blocks and flags in which 50% of quartz in the finish–visible layer has been replaced by panel recycled cathode ray tube glass. The review of literature did not reveal any paper discussing this topic, which provides a certain originality to our paper. The main goal set for this research is proving the potential use of panel CRT recycled glass as a partial replacement for natural aggregate in the finish layer of concrete blocks and paving flags, which is a possible solution for disposal. We focus on the analysis of properties that relate the most to the finish layer of the paving elements. Within the experimental research, physical and mechanical properties as well as the durability of concrete are tested: shape and dimensions, splitting tensile strength, tensile strength by bending, water absorption, resistance to freeze/thaw and de-icing salts, wear resistance (Böhme test), slip resistance (SRT pendulum test), radioactivity and leaching. For the purpose of observing the concrete microstructure, SEM and EDS analyses were performed in order to acquire the knowledge of the concrete surface.

## 2. Materials and Methods

### 2.1. Materials

For the production of concrete blocks, cement CRH Serbia CEM I 52.5R was used, which meets all the quality requirements of SRPS EN 197-1:2013. Physico-mechanical cement characteristics are presented in Table 1. The chemical composition of cement is shown in Table 2.

**Figure 2:**
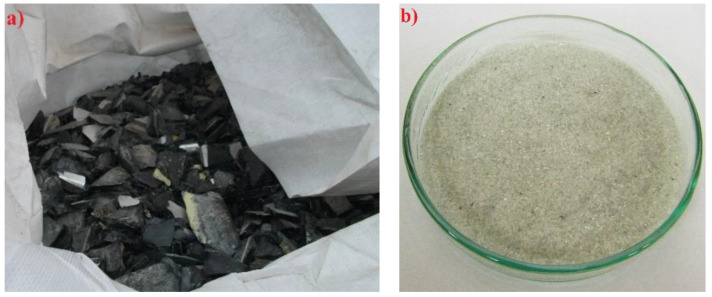
Shards of glass after recycling (**a**) and CRT glass 0.25/1.00 mm (**b**).

**Figure 3:**
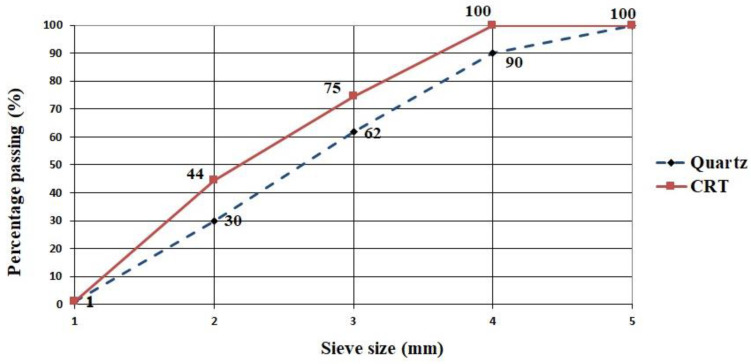
Particle size distribution of quartz aggregate and panel CRT glass.

The glass used in the experiment was panel CRT glass obtained by recycling old TV sets and other electronic devices. Large shards of glass (Figure 2a) were ground to the fineness 0–4 mm in the local asphalt plant. Further grinding of glass to the fraction 0.25–1.0 mm (Figure 2b) was performed in the laboratory using a ball mill. This fraction was used for making the visible layer of concrete blocks. The visible layer can be made in any color; we used a mineral pigment in black color (in accordance with EN 12878), namely Bayferrox 330 manufactured by Bayer Germany. This pigment is a powder, and its chemical composition is synthetic iron oxide Fe_3_O_4_. The chemical composition of cathode ray tube glass is presented in Table 2. The particle size distributions of CRT glass and quartz aggregates are shown in Figure 3.

**Figure 4:**
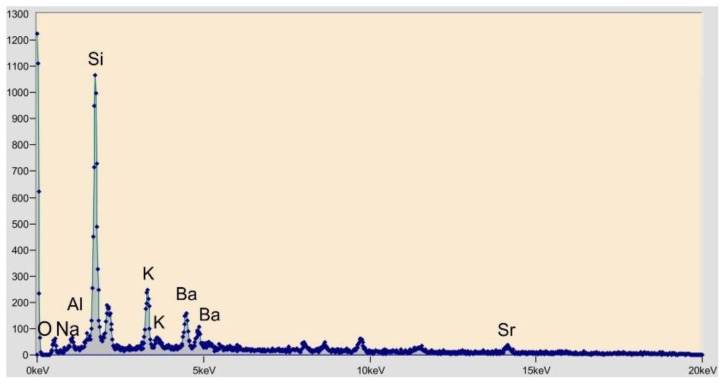
EDS spectrometry of cathode ray tube glass.

**Figure 5:**
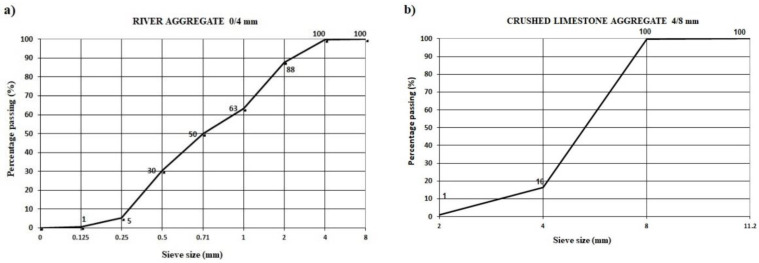
Particle size distribution of river aggregate 0/4 mm (**a**) and crushed limestone aggregate 4/8 mm (**b**).

EDS spectroscopy of the ground CRT glass (Figure 4) determined a considerable presence of silicon. Oxygen, sodium, aluminum and potassium were also present. Because that the panel glass was coated with protective layers during the service life of the electronic device, the EDS spectroscopy detected the presence of barium and strontium in traces, which originate from these protective layers. The basic concrete layer was made with river aggregate fraction 0/4 mm (Figure 5a) and crushed limestone aggregate fraction 4/8 mm (Figure 5b). For the finishing layer, the visible layer was quartz sand with a particle size 0.25–1.2 mm. Physical properties of used aggregates are provided in Table 3.

### 2.2. Mix Design

Two concrete mix designs were developed, and their compositions are shown in Table 4. In the case of the reference series (E), the finishing layer of concrete blocks was made with 100% quartz, while in the mixture marked with WG, 50% of quartz was replaced by 0.25/1.00 mm cathode ray tube glass.

**Figure 6:**
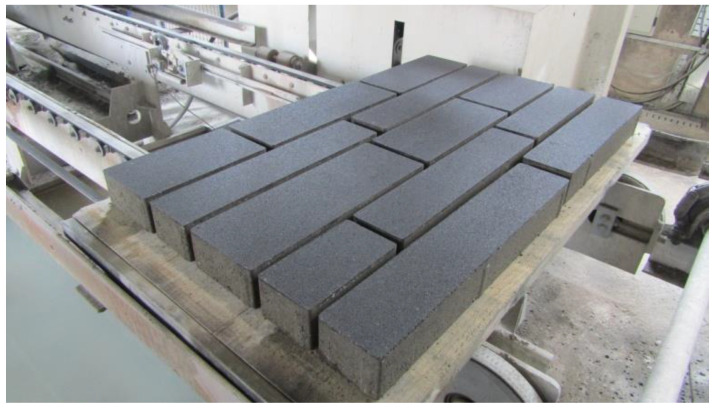
Appearance of concrete blocks and flags.

The production process of concrete blocks and flags is fully automated and computerized. Depending on the dimensions, the product is categorized as a block or flag (EN 1338 [25], EN 1339 [26]). In the experiment, the base layer of concrete was compacted by simultaneous vibration and pressing. In the second step, the components of the final visible layer of concrete blocks and flags were dosed, and the process of vibration and pressing was repeated once again. Blocks measuring 210 × 115 × 80 mm (16 specimens) and 300 × 165 × 80 mm (32 specimens) were produced, as well as flags measuring 390 × 165 × 80 mm (32 specimens), 420 × 115 × 80 mm (16 specimens), 480 × 165 × 80 mm (32 specimens), 540 × 115 × 80 mm (48 specimens) and 630 × 115 × 80 mm (32 specimens), for a total of 208 specimens. The same number of specimens was made without glass (reference specimens). The final visible layer of samples was on average 8 mm thick over the entire surface of the samples. The upper visible side of the samples was flat without curvature (Figure 6).

### 2.3. Test Methods

The following tests were performed on the concrete blocks:

- Shape and dimensions (EN 1338, annex C);
- Resistance to freeze/thaw and de-icing salts (EN 1338, annex D);
- Water absorption (EN 1338, annex E);
- Splitting tensile strength (EN 1338, annex F);
- Tensile strength by bending (EN 1339, annex F);
- Wear resistance—Böhme test (EN 1338, annex H);
- Slip resistance—SRT pendulum (EN 1338, annex I);
- Radioactivity testing;
- Leaching test; and
- SEM concrete analysis.

The testing principle of freeze/thaw resistance and de-icing salts is based on the procedure in which the prepared samples are submitted to 28 freeze/thaw cycles while the surface is covered with a 3% NaCl solution. The scaled off material was collected and weighed, and the result is expressed in kilograms per square meter. The number of tested samples was 6 of E concrete and 6 of WG concrete. The tested surface area of each sample was 22,500 mm^2^. The testing was performed in the Climatic Cabinet Controls 10-D1428/A with the time temperature cycle prescribed by the standard EN 1338, annex D. The mean values of the test results are provided in Table 5.

Total water absorption was tested on 10 samples of E concrete and WG concrete according to the standard EN 1338, annex E. After conditioning the specimen to (20 ± 5) °C, it was soaked to constant mass and then oven-dried to constant mass. The loss in mass is expressed as a percentage of the mass of the dry specimen. The mean values of the test results are provided in Table 5.

**Figure 7:**
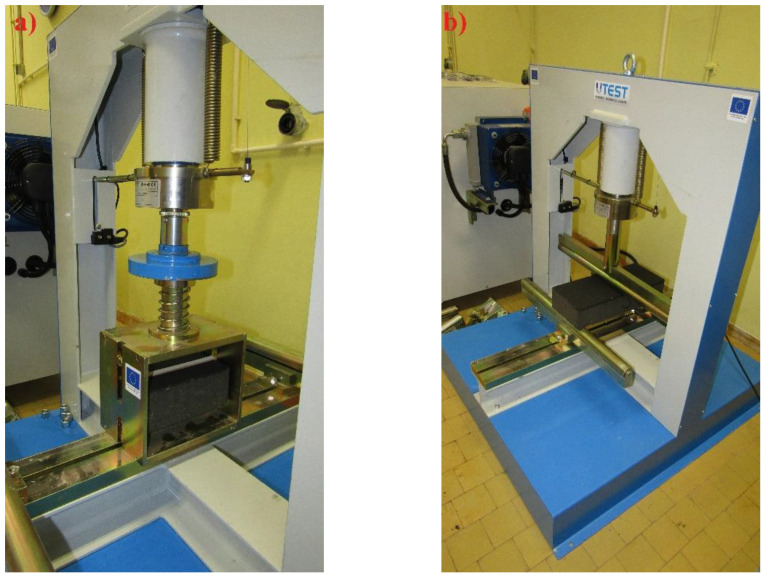
Disposition when testing the splitting tensile strength (**a**) and the tensile strength by bending test (**b**).

Splitting tensile strength tests were conducted on concrete blocks having 210 × 115 × 80 mm dimensions according to the EN 1338 standard, annex F. The upper and lower surfaces of the block were ground to smoothness. The tests were conducted on a digital hydraulic press UTEST UTC—5600 (Figure 7a). There were regular stress increments of 0.05 MPa up to failure of the specimen, and the load was applied by the packing pieces.

Tensile strength by bending tests were conducted on concrete flags having 390 × 165 × 80 mm dimensions according to the EN 1339 standard, annex F. The flags were ground at their support points to achieve as satisfactory contact as possible. The load increments were such that failure was achieved after 45 s ± 15 s from the start of the load application (Figure 7b).

Measuring of abrasion according to the Böhme test was performed on 6 cube-shaped samples with 71 mm sides on the Böhme Machine Controls 48-D5272. The samples were placed on the Böhme disc abrader, the test track on which standard abrasive is strewn, and the disc was rotated and the specimens were subjected to an abrasive load of (294 ± 3) N for 16 cycles. The mean loss in specimen volume ΔV after 16 cycles of testing in mm^3^/mm^2^ is provided in Table 5.

The measurement of USRV on the specimen was made using the pendulum friction test equipment to evaluate the frictional properties of the specimen on the upper face. The pendulum friction test equipment (Portable Skid Resistance Tester, Stanley London) incorporates a spring-loaded slider made of a standard rubber attached to the end of the pendulum. Upon swinging the pendulum, the frictional force between the slider and test surface was measured by the reduction in length of the swing using a calibrated scale.

In order to confirm of the possibility of using concrete blocks and flags made with recycled glass, the radioactivity of the concrete in question was checked. The reference level for outdoor irradiation in an enclosed space originating from natural gamma emitters from building materials, excluding the outdoor irradiation in the open air, is E = 1 mSv a year, where E is the effective dose and the unit is Sievert (1 Sv = 1 J kg^−1^). In building materials, the contents of the following radionuclide elements are determined: Ra—226, Th—232 (or Ra—228) and K—40. Based on the obtained values, the gamma index is calculated according to the equation:

(1) $$I=\frac{C_{\mathrm{Ra}226}}{300\mathrm{Bq}/\mathrm{kg}}+\frac{C_{\mathrm{Th}232}}{200\mathrm{Bq}/\mathrm{kg}}+\frac{C_{K40}}{3000\mathrm{Bq}/\mathrm{kg}}\;$$

where:

C_Ra_ is the concentration of radium (^226^Ra) in Bq/kg;
C_Th_ is the concentration of thorium (^232^Th) in Bq/kg; and
C_K_ is the concentration of potassium (^40^K) in Bq/kg.

The gamma index was derived based on the effective dose criterion, *E* < 1 mSv, and the concentration of activities in different materials. For use in the construction industry, the gamma index should be <1.

**Figure 8:**
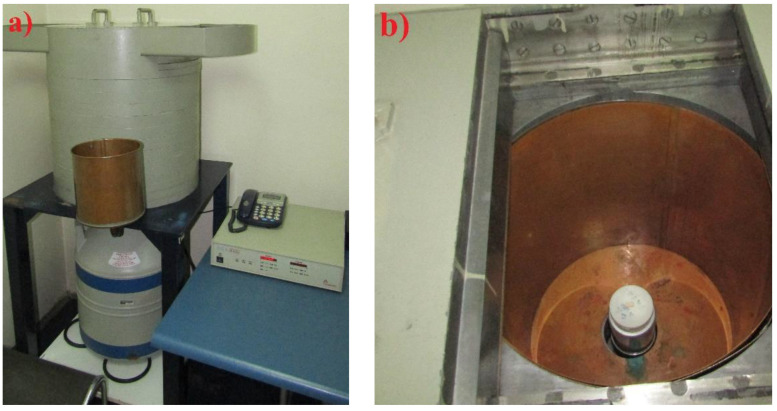
HPGe detector linked to a Canberra DSA-2000 Multichannel Analyzer coupled to the Genie 2000 software (**a**) and sample placed in the shield (**b**).

The radioactivity tests were performed on the pure CRT glass (3 samples), reference concrete (3 samples) and 3 samples of WG concrete. Samples of reference concrete and concrete with recycled glass (WG) were ground to pass the sieve with a mesh size of 1 mm without residue, and they were used as such in the tests. Gamma spectrometric analysis was performed using the HPGe gamma spectrometer Canberra DSA—2000 (Figure 8). An HPGe (high-purity germanium) detector was used with 25% efficiency and 1.85 keV FWHM (full width at half maximum) at 1332 keV (Co-60). Ra-226 activity concentrations were detected and measured on progenies of Pb-214 and Bi-214 at energies of 609 KeV, 295 keV and 351 keV. K-40 activity concentrations were measured at energy of 1460 keV. Th-232 activity concentration was measured at photopeak energy of 911 keV of Ac-228 as a first progeny of Th-232 after its alpha decay. The laboratory of Institute of Occupational Health in Niš, Serbia, where measurements were performed, is accredited in accordance with the ISO 17025 standard. The samples were placed in a 100 mL utensil. It is very important to accurately measure the mass of the sample before testing so that after the measurement, it is possible to calculate the presence of radionuclides in Bq per 1 kg. Becquerel is defined as the activity of the amount of radioactive material, with one Becquerel corresponding to one decay per second: 1 Bq = 1 s^−1^. Moreover, since different half-lives are required for different atomic nuclei, it is desirable to perform the test for several hours in order to detect all half-lives. The spectrometer used contains a hyper-pure germanium semiconductor detector and is fully digitized so that the measured values of gamma radiation are generated and displayed using the “Genie 2000” computer software. The complete test procedure is prescribed by the International Atomic Energy Agency in IAEA TRS 295: 1989.

In order to determine the impact of the addition of CRT glass on the environment, the leaching test was performed. This test examines the impact of potentially hazardous chemical compounds present in the finishing layer of concrete on the quality of ground waters, and determines if there is a potential risk for human health. The eluates for the reference concrete and WG concrete were obtained according to the standard EN 1744-3 [27]. The chemical composition of the eluates was determined at the Laboratory for General and Inorganic Chemistry of the Faculty of Science and Mathematics of Niš.

The SEM analysis was conducted for the purpose of assessing the texture of the surface of the finish layer of reference concrete and of concrete with a modified finish layer.

## 3. Results and Discussion

The test results for the concrete paving blocks and flags are shown in Table 5. Unfortunately, some of the results presented in Table 5 cannot be compared to the results of other authors because CRT glass was not used for modification of the visible layer of concrete blocks and paving flags.

**Figure 9:**
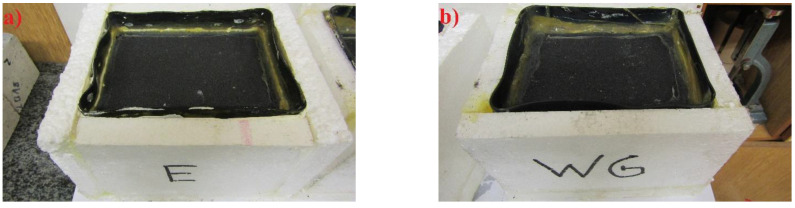
The appearance of the surfaces of reference concrete blocks (**a**) and concrete blocks modified with CRT glass (**b**) after 28 cycles of freeze/thaw and de-icing salt action.

Based on the obtained results, it is clear that the replacement of 50% quartz with panel cathode ray tube glass only in the visible layer of concrete samples does not cause a change in density. The amount of panel CRT glass used for replacing quartz sand in 1 m^3^ of concrete (basic + finishing layer) was 74 kg. The absence of a change in density is explained by the similar values of densities of quartz sand (2.65 g/cm^3^) and panel CRT glass (2.84 g/cm^3^), and of their low content in 1 m^3^ of concrete. After 28 cycles of simultaneous action of frost and defrosting salt, there was no damage to the concrete surface (surface scaling), neither in the reference series (Figure 9a) nor in the series with panel cathode ray tube glass (Figure 9b).

The research by Lee et al. [18] shows that the increase in the replacement of natural aggregate with recycled glass causes an increase in water absorption of concrete blocks. It was determined that this increase is related to the particle size distribution and that the finer the glass is ground, the higher the water absorption increase. The absorption of water in both experimental series was uniform—5% on average. This can be explained by the fact that the share of panel CRT glass is small, as little as 3.4% in the total mass of concrete (basic + finishing layer). Moreover, the particle size distribution of CRT glass was very similar to the replaced quartz aggregate (Figure 3). According to the standard EN 1338, the stipulated quality condition is that the concrete blocks must have splitting tensile strength of no less than 3.6 MPa. The results of these tests range between 3.73 MPa and 4.14 MPa, so both series of concrete blocks meet the prescribed quality condition. According to the standard EN 1339, the stipulated quality condition is that the concrete flags must have tensile strength by bending of no less than 3.5 MPa. In the above test, the obtained bending tensile strength tests range between 3.78 MPa and 4.02 MPa, so both experimental series meet the quality condition prescribed by the standard. On the basis of the abrasion wear resistance test results, one can observe the considerable difference between the samples with and without the recycled glass in the finishing layer, since the concrete blocks with 50% CRT glass had 26.42% lower abrasion wear value than the reference series. The explanation for such an effect of the replacement of a part of quartz sand with glass in the finish layer of concrete blocks and flags should be a subject of deeper analysis, which would include an analysis of the interstitial transit zone (ITZ) between the aggregate grains (quartz and CRT glass) and the cement matrix.

The SRT pendulum test demonstrated that the presence of glass in the finishing layer only negligibly reduces the slip resistance of concrete blocks. At any rate, the slip resistance can be characterized as extremely low, which makes the concrete blocks a suitable surface for pedestrian traffic.

Measured values of radioactivity of pure CRT glass, reference concrete and concrete with recycled glass WG are presented in Table 6.

The gamma index (I) of concentrated glass amounts to 1.076 (>1). According to the “Regulations on the limit values of radionuclide for drinking water, various food items, building materials and other commodities”, it is necessary to calculate the level of an effective dose, which is used to assess the application and conditions of use of materials in the construction industry. In the cases of the reference concrete and the concrete with recycled glass, the gamma index is uniform, amounting to 0.295 and 0.359, respectively. The measured values are considerably lower than the limits, so the presence of recycled panel cathode ray tube glass does not have an adverse effect on the environment and human health in terms of increased radioactivity emitted by CRT glass.

The results of the leaching test are shown in Table 7. The metals most commonly reported as hazardous or toxic in different types of sediments or materials were selected for the presentation. It can be seen that all concentration values of such metals are less than 1 mg/kg, which is far below the limit values prescribed by the “Regulations on the permissible quantities of hazardous and harmful substances in soil and irrigation water” [28]. Cadmium, lead and mercury, as the most prominent representatives of hazardous substances, are also within the permissible limits. In 1 m^3^ WG concrete (basic + finishing layer), there is around 74 kg of panel CRT glass aggregate. For such a concrete mixture, the cathode ray tube glass has a mass share of around 3.4%. The obtained results of the chemical analysis of the eluate of WG concrete unequivocally indicate that it presents no danger to the environment and human health.

**Figure 10:**
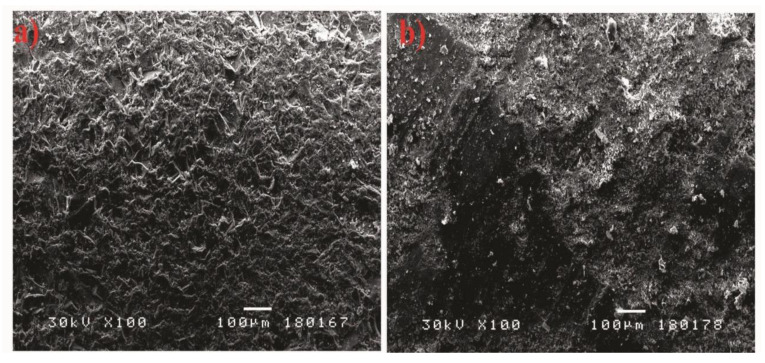
Reference concrete sample (**a**) and WG concrete sample (**b**) at 100× magnification.

The texture of the reference concrete is compact and homogeneous (Figure 10a). The texture of the WG concrete (Figure 10b) is also homogeneous, with the surface completely flat across most of the sample.

## 4. Conclusions

The replacement of 50% of quartz sand with panel recycled glass in the finishing layer of concrete paving blocks and flags does not cause a change in bulk density, nor does it impair the durability of the product in terms of simultaneous action of frost and de-icing salt. Additionally, it does not increase water absorption or reduce tensile splitting strength and tensile strength by bending below the values prescribed by standards EN 1338 and EN 1339. Furthermore, the presence of recycled glass slightly reduced the slip resistance of concrete blocks, while the resistance to wear by Böhme grinding was significantly improved. It is our opinion that in future research, it would be interesting to perform a deeper SEM analysis which would include the interstitial transit zone (ITZ) between the aggregate grains and the cement matrix.

The test of radioactivity has unequivocally shown that the use of recycled cathode glass in concrete does not increase the radiation dose above the limit that could adversely affect the environment and human health.

All concentration values of potentially hazardous chemical compounds in eluates obtained by the leaching test are far less than 1 mg/kg, which is far below the limit values prescribed by regulations [28]. The chemical analysis of WG concrete indicates that it presents no danger to the environment and human health.

Recycled cathode glass can be successfully used for the production of concrete blocks and paving flags, representing part of the solution of the problem of existing landfills and a contribution to sustainable construction. The possible use of waste CRT glass presented in this paper is considerable, considering that the closed loop of recycling, which comprises the use of old CRT screens for the production of new ones, is not an option any longer.

Panel glass constitutes around two-thirds of one CRT screen, or around 10 kg [29]. The surface area of produced concrete blocks and flags in Serbia in 2021 was ca. 1,200,000 m^2^. If for the production of the finish layer, panel CRT aggregate is used to replace 50% of quartz sand mass, which amounts to 10 kg/1 m^2^ (as in this paper), then the entire production would require 12,000 tons of glass. Even if it is acknowledged that not all concrete elements have two layers, it can be concluded that the entire collected amount of panel CRT glass can be consumed in this way. The cost of quartz sand in Serbia is around 5 euro cents per 1 kg. The costs of collecting old CRT devices, the separation of panel and funnel glass and crushing is 30 euro cents per 1 kg, which is around six times higher than the quartz sand cost. However, the cost of the reuse, recycling and utilization of CRT screens as a raw material amounts to 73 euro cents per 1 kg [30]. Having in mind the mentioned costs, concrete makers are incentivized to use CRT glass as a component in the production of concrete and concrete products.

One of the problems that must be solved in the future is forming legislative regulations and standards which would coordinate the classification of CRT glass and recycling and waste disposal solutions.

## Figures and Tables

**Table 1:** Physico-mechanical characteristics of cement CEM I 52.5R, “CRH” Novi Popovac, Serbia.

Characteristic	Result	Quality Requirement SRPS EN 197-1	Quality Assessment
Standard consistency (%)	29.6	max. 31–32%	satisfies
Initial setting time (min)	161	≥50 min	satisfies
Soundness (mm)	0.0	max. 10 mm	satisfies
Sieve residue	0.0	max. 10%	satisfies
Specific gravity (g/cm^3^)	3.15	min. 3.0	satisfies
Blaine spec. surf. (m^2^/kg)	394	min. 2400	satisfies
Flexural strength (MPa)	5.7 (2 days)	not prescribed	
	8.5 (28 days)		
Compressive strength (MPa)	33.5 (2 days)	min. 20 MPa	satisfies
	59.5 (28 days)	min. 52.5 MPa	satisfies

**Table 2:** Chemical composition of CRT glass and cement.

Chemical Compound	SiO_2_ (%)	Al_2_O_3_ (%)	Fe_2_O_3_ (%)	CaO (%)	MgO (%)	K_2_O (%)	Na_2_O (%)	SO_3_ (%)	LOI (%)	Density kg/m^3^
CRT glass	60.61	2.88	0.58	1.31	0.53	6.45	7.61	0.09	1.04	2840
Cement	19.30	4.28	2.87	62.8	2.22	0.91	0.21	3.05	2.26	3150

**Table 3:** Physical properties of fine aggregates and coarse aggregates.

Physical Properties	River Aggregate	Crushed Limestone Aggregate	Quartz
	0/4 mm	4/8 mm	0.25–1.2 mm
Relative density SSD * (kg/m^3^)	2610	2690	2600
Apparent specific gravity (kg/m^3^)	2640	2730	2650
Water absorption (%)	1.42	0.95	1.16
Shape	Rounded	Angular	Angular

* SSD—density at saturated surface dry condition.

**Table 4:** Composition of concrete mixtures.

Concrete	Block Layer	Volume of Concrete	Aggregate 0/4 mm	Aggregate 4/8 mm	Cement	Quartz 0.25/1.2 mm	CRT 0.25/1.0 mm	Water	Black Pigment
		m^3^	kg	kg	Kg	kg	kg	kg	kg
E	Basic	0.25	280	220	95	-	-	36	-
	Finishing	0.0278	-	-	20	37	-	7.5	1.1
WG	Basic	0.25	280	220	95	-	-	36	-
	Finishing	0.0278	-	-	20	18.5	18.5	7.5	1.1

**Table 5:** Test results of concrete paving blocks and flags.

Test	E	WG	Quality Condition According to EN 1338/1339
Density (kg/m^3^)	2172	2174	-
Resistance to freeze/thaw and de-icing salts (kg/m^2^)	No damage	No damage	≤1.0
Water absorption (%)	5.06	5.12	≤6.0
Splitting tensile strength (MPa)	4.14	3.73	≥3.6
Tensile strength by bending (MPa)	4.02	3.78	≥3.5
Wear abrasion test Böhme method (mm^3^/mm^2^)	18,585	13,675	≤20,000 mm^3^/mm^2^ ≤18,000 mm^3^/mm^2^
Slip resistance (USRV ^1^)	147.5	136.5	Not prescribed

^1^ USRV—unpolished slip resistance.

**Table 6:** Concentrations of radionuclide activity.

Material	Concentration of Radionuclide Activity			Gamma Index
	C_Ra226_	C_Th232_	C_K40_	I
	Bq/kg	Bq/kg	Bq/kg	-
CRT glass (pure)	76 ± 6	36 ± 3	1928 ± 157	1.076
Reference concrete	21 ± 2	19 ± 2	389 ± 33	0.295
Concrete WG	25 ± 2	21 ± 2	512 ± 43	0.359

**Table 7:** Contents of potentially hazardous metals in concrete eluates.

Concrete Eluate/Metal Oxides	Cr	Mn	Co	Ni	Cu	Zn	Cd	Ba	Pb	Hg
	(mg/kg)									
E	<0.01	0.01	0.02	0.01	0.02	0.02	<0.01	0.33	0.02	n.d. *
Concrete WG	0.01	0.02	0.03	0.06	0.06	0.18	0.02	0.13	0.05	n.d. *

* Not detected.

## Data Availability

Publicly available datasets were analyzed in this study. This data can be found here: [https://nardus.mpn.gov.rs/handle/123456789/18135?fbclid=IwAR3VMPCrUU8da8atlDzwnMuxbpiL5zX2iAxg2I7VK-Fj-ZpeuIMe1GADN14] (accessed on 10 February 2022).

## References

[1] International Association of Electronic Waste Producer Responsibility Organisations (WEEEforum) Impact of Glass from Cathode Ray Tubes (CRT) in Achieving the WEEE Recycling and Recovery Targets, BluePoint Conference and Business Centre, Bouleverd Auguste Reyerslaan 80 B-1030 Brussels, Belgium. https://weee-forum.org/wp-content/uploads/2019/06/CRT-glass_Issue-paper_Final.pdf.

[2] Pauzi N.N.M., Shah M.S.A. (2020). Durability Properties of Concrete Containing Waste Cathode Ray Tube Glass as Fine Aggregates—A Review. J. Environ. Treat. Tech..

[3] Xu Q., Li G., He W., Huang J., Shi X. (2012). Cathode ray tube (CRT) recycling: Current capabilities in China and research progress. Waste Manag..

[4] Singh N., Tang Y., Li J. (2019). Uncovering material flow analysis of waste cathode ray tubes television in China. Waste Manag. Res..

[5] Grdić D., Topličić-Ćurčić G., Grdić Z., Ristić N. (2021). Durability Properties of Concrete Supplemented with Recycled CRT Glass as Cementitious Material. Materials.

[6] Mear F., Yot P., Cambon M., Ribes M. (2006). The characterization of waste cathode-ray tube glass. Waste Manag..

[7] Singh N., Li J., Zeng X. (2016). Solutions and challenges in recycling waste cathode-ray tubes. J. Clean. Prod..

[8] Alex X.I., Arunachalam K. (2019). Flexural behavior of filer reinforced lightweight concrete. Rev. Conctr..

[9] Cordero B. (2015). Thermal performance of novel frame integrated unitised curtain wall. Rev. Conctr..

[10] Zhao H., Wei S. (2011). Study of properties of mortar containing cathode ray tubes (CRT) glass as replacement for river sand fine aggregate. Const. Build. Mater..

[11] Ling T.C., Poon C.S. (2011). Utilization of recycled glass derived from cathode ray tube glass as fine aggregate in cement mortar. J. Hazard. Mater..

[12] Bignozzi M.C., Saccani A., Barbieri L., Lancelloti I. (2014). Glass waste as supplementary cementing materials: The effects of glass chemical composition. Cem. Concr. Compos..

[13] Aliabdo A.A., Elmoaty A., Aboshama A. (2016). Utilization of waste glass powder in the production of cement and concrete. Const. Build. Mater..

[14] Lu J.X., Duan Z.H., Poon C.S. (2017). Fresh properties of cement pastes or mortars incorporating waste glass powder and cullet. Const. Build. Mater..

[15] Grdić D., Ristić N., Topličić-Ćurčić G., Đorđević D., Krstić D. (2020). Effects of addition of finely ground CRT glass on the properties of cement paste and mortar. Građevinar.

[16] Cabrera M., Pérez P., Rosales J., Agrela F. (2020). Feasible Use of Cathode Ray Tube Glass (CRT) and Recycled Aggregates as Unbound and Cement-Treated Granular Materials for Road Sub-Bases. Materials.

[17] Zhao H., Poon C.S., Ling T.C. (2013). Properties of mortar prepared with recycled cathode ray tube funnel glass sand at different mineral admixture. Constr. Build. Mater..

[18] Lee G., Poon C.S., Wong Y.L., Ling T.C. (2013). Effect of recycled fine glass aggregates on the properties of dry-mixed concrete blocks. Constr. Build. Mater..

[19] Ganjian E., Jalull G., Sadeghi-Pouya H. (2015). Using waste materials and by—Products to reduce concrete paving blocks. Constr. Build. Mater..

[20] Dondi D., Guarini G., Raimondo M., Zaneli C. (2009). Recycling PC and TV waste glass in clay bricks and roof flags. Waste Manag..

[21] Andreola F., Barbieri L., Corradi A., Lancellotti I. (2007). CRT glass state of the art: A case study: Recycling in ceramic glazes. J. Eur. Ceram. Soc..

[22] Zhang Q., He F., Shu H., Qiao Y., Mei S., Jin M., Xie J. (2016). Preparation of high strength glass ceramic foams from waste cathode ray tube and germanium tailings. Constr. Build. Mater..

[23] Yu M., Liu L., Li J. (2016). An overall solution to cathode-ray tube (CRT) glass recycling. Procedia Environ. Sci..

[24] Ling T.C., Poon C.S., Lam W.S., Chan T.P., Fung K.K. (2012). Utilization of recycled cathode ray tubes glass in cement mortar for X-ray radiation—Shielding applications. J. Hazard. Mater..

[25] (2012). Concrete Paving Blocks—Requirements and Test Methods.

[26] (2008). Concrete Paving Flags—Requirements and Test Methods.

[27] (2007). Tests for Chemical Properties of Aggregates—Part 3: Preparation of Eluates by Leaching of Aggregates.

[28] (1994). Regulations on the permissible quantities of hazardous and harmful substances in soil and irrigation water. Official Gazette of RS.

[29] Singh N., Wang J., Li J. (2016). Waste cathode rays tube: An assessment of global demand for processing. The Tenth International Conference on Waste Management and Technology (ICWMT). Procedia Environ. Sci..

[30] Pokimica N., Nedeljković-Bunardžić K., Krstović S. (2018). Analysis of the State of Electrical and Electronic Waste Management in the Republic of Serbia.

